# From the Cochrane Library: Interventions for Preventing Occupational Irritant Hand Dermatitis

**DOI:** 10.2196/37961

**Published:** 2022-07-06

**Authors:** Sara Abdel Azim, Natalia Vecerek, Alex G Ortega-Loayza, Andrea Bauer, Brandon L Adler

**Affiliations:** 1 Georgetown University School of Medicine Washington, DC United States; 2 Department of Dermatology Keck School of Medicine University of Southern California Los Angeles, CA United States; 3 Department of Dermatology Oregon Health and Science University Portland, OR United States; 4 Department of Dermatology University Hospital Carl Gustav Carus Technical University Dresden Dresden Germany

**Keywords:** occupational irritant hand dermatitis, barrier creams, moisturizers, skin protection education, protective gloves, prevention, COVID-19, Cochrane, meta-analysis, irritant, dermatitis, hand, skin, dermatology, occupational, skin disease, prevention, protection

Occupational hand dermatitis, the most common work-related skin disease, is divided into irritant and allergic types [1]. Occupational irritant hand dermatitis (OIHD) is associated with repetitive wet work, contact with detergents and other chemicals, and prolonged glove wearing. OIHD frequently becomes chronic, exerts a major impact on quality of life, and may eventuate in disability or job loss/change. As such, its prevention is paramount.

In this paper, we summarize findings from a 2018 Cochrane systematic review and meta-analysis assessing the efficacy of strategies for primary prevention of OIHD [2]. Inclusion criteria specified randomized controlled trials (RCTs) of barrier creams, moisturizers, gloves, or educational programs involving employees without pre-existing OIHD working in high-risk fields. Databases were searched without language restriction through the end of January 2018. The primary outcomes were incidence of new-onset OIHD and frequency of intervention discontinuation owing to adverse effects.

In total, 9 RCTs were included, all conducted in Europe except for 1 from Singapore. The 2888 participants consisted of metalworkers, factory and slaughterhouse workers, cleaners and kitchen workers, hospital employees, and hairdressing apprentices, who ranged in age from 16 to 67 years. Interventions included barrier creams, moisturizers, barrier creams combined with moisturizers, and educational programs; no studies investigated protective gloves. The mean duration of the intervention was 11.6 months. Meta-analysis revealed that for all interventions, fewer participants developed OIHD compared to controls (Table 1); however, the differences were not statistically significant. The pooled analyses showed wide CIs, and the studies may not have been adequately powered to detect differences. None of the studies addressed the frequency of discontinuation of the intervention relating to adverse effects; however, recorded dropout reasons were unrelated to adverse effects; therefore, these strategies likely cause few or no serious side effects.

**Table 1:** Effects of interventions on development of occupational irritant hand dermatitis.

Intervention	Studies, n	Follow-up (months)	Participants, n	Proportion of participants developing OIHD^a^ (%)		Relative effect, RR^b^ (95% CI)	Quality of evidence^c^
				Patients	Controls		
Barrier creams^d^	4	6-12	999	29	33	0.87 (0.72-1.06)	Low
Moisturizers^e^	3	6-12	507	13	19	0.71 (0.46-1.09)	Low
Barrier creams + moisturizers	2	12 (median)	474	8	13	0.68 (0.33-1.42)	Low
Skin protection education	3	12-36	1355	21	28	0.76 (0.54-1.08)	Very low

^a^OIHD: occupational irritant hand dermatitis.

^b^RR: risk ratio

^c^Evidence assessed using Grading of Recommendations, Assessment, Development and Evaluation Working Group criteria [3].

^d^Examples of barrier creams used include Arretil, Ache Basis Creme, Excipial, Stoko Protect, and Travabon.

^e^Examples of moisturizers used include Estolan, Keri Lotion, and Locobase.

There are several potential limitations of this Cochrane review. It included a small number of trials, mainly conducted in Europe, that used heterogeneous diagnostic criteria for OIHD. Additionally, no studies were designed to exclude patients with endogenous/atopic or allergic hand eczema (through patch testing). The ability to compare studies was limited due to variations in follow-up time and the nature of included occupations. Overall, the quality of the evidence was judged to be low.

This Cochrane review found that barrier creams and moisturizers may reduce the risk of developing OIHD to some degree, but there was insufficient evidence to support the effectiveness of the evaluated workplace interventions in the primary prevention of OIHD. This does not imply that these interventions are *not* effective; on the contrary, barrier creams, moisturizers, and gloves continue to be broadly recommended as crucial measures for occupational skin protection, particularly in the current era of increased hand hygiene requirements during the SARS-CoV-2 (COVID-19) pandemic [4]. An important consideration is that suboptimal real-world use of prevention strategies may fail to demonstrate the efficacy observed in experimental settings [5]. To reach more certain conclusions, there remains a need for large and pragmatic worldwide RCTs using uniform inclusion and diagnostic criteria for OIHD conducted over extended follow-up periods (6-12+ months).

## Abbreviations

OIHD: occupational irritant hand dermatitis
RCT: randomized controlled trial

## References

[1] Diepgen TL, Coenraads PJ (1999). The epidemiology of occupational contact dermatitis. Int Arch Occup Environ Health.

[2] Bauer A, Rönsch H, Elsner P, Dittmar D, Bennett C, Schuttelaar MLA, Lukács J, John SM, Williams HC (2018). Interventions for preventing occupational irritant hand dermatitis. Cochrane Database Syst Rev.

[3] Schunemann H, Brozek J, Guyatt G, Oxman A (2013). GRADE handbook for grading quality of evidence and strength of recommendations. GRADE Working Group.

[4] Rundle CW, Presley CL, Militello M, Barber C, Powell DL, Jacob SE, Atwater AR, Watsky KL, Yu J, Dunnick CA (2020). Hand hygiene during COVID-19: recommendations from the American Contact Dermatitis Society. J Am Acad Dermatol.

[5] Schliemann S, Petri M, Elsner P (2012). How much skin protection cream is actually applied in the workplace? Determination of dose per skin surface area in nurses. Contact Dermatitis.

